# Analysis of Line and Tube Detection Performance of a Chest X-ray Deep Learning Model to Evaluate Hidden Stratification

**DOI:** 10.3390/diagnostics13142317

**Published:** 2023-07-09

**Authors:** Cyril H. M. Tang, Jarrel C. Y. Seah, Hassan K. Ahmad, Michael R. Milne, Jeffrey B. Wardman, Quinlan D. Buchlak, Nazanin Esmaili, John F. Lambert, Catherine M. Jones

**Affiliations:** 1 Annalise.ai, Sydney, NSW 2000, Australia; 2 Intensive Care Unit, Gosford Hospital, Sydney, NSW 2250, Australia; 3 Department of Radiology, Alfred Health, Melbourne, VIC 3004, Australia; 4 School of Medicine, The University of Notre Dame Australia, Sydney, NSW 2007, Australia; 5 Department of Neurosurgery, Monash Health, Melbourne, VIC 3168, Australia; 6 Faculty of Engineering and Information Technology, University of Technology Sydney, Ultimo, NSW 2007, Australia; 7 I-MED Radiology Network, Brisbane, QLD 4006, Australia; 8 School of Public and Preventive Health, Monash University, Clayton, VIC 3800, Australia; 9 Department of Clinical Imaging Science, University of Sydney, Sydney, NSW 2006, Australia

**Keywords:** machine learning, chest X-ray, deep learning, hidden stratification, lines and tubes

## Abstract

This retrospective case-control study evaluated the diagnostic performance of a commercially available chest radiography deep convolutional neural network (DCNN) in identifying the presence and position of central venous catheters, enteric tubes, and endotracheal tubes, in addition to a subgroup analysis of different types of lines/tubes. A held-out test dataset of 2568 studies was sourced from community radiology clinics and hospitals in Australia and the USA, and was then ground-truth labelled for the presence, position, and type of line or tube from the consensus of a thoracic specialist radiologist and an intensive care clinician. DCNN model performance for identifying and assessing the positioning of central venous catheters, enteric tubes, and endotracheal tubes over the entire dataset, as well as within each subgroup, was evaluated. The area under the receiver operating characteristic curve (AUC) was assessed. The DCNN algorithm displayed high performance in detecting the presence of lines and tubes in the test dataset with AUCs > 0.99, and good position classification performance over a subpopulation of ground truth positive cases with AUCs of 0.86–0.91. The subgroup analysis showed that model performance was robust across the various subtypes of lines or tubes, although position classification performance of peripherally inserted central catheters was relatively lower. Our findings indicated that the DCNN algorithm performed well in the detection and position classification of lines and tubes, supporting its use as an assistant for clinicians. Further work is required to evaluate performance in rarer scenarios, as well as in less common subgroups.

## 1. Introduction

The insertion of support devices, such as endotracheal tubes (ETTs), enteric tubes (NGTs), and central venous catheters (CVCs), is a common procedure in hospitalised patients to facilitate provision of care in the acute setting. However, complications resulting from malpositioning of such devices can lead to significant morbidity and mortality, due to an inability to deliver treatment or through direct harm from the insertion procedure [1,2,3]. For example, it has been reported that, in England and Wales between 2005 and 2010, there were 21 deaths and 79 cases of harm due to misplaced NGTs [4]. In many health jurisdictions, the utilisation of inadvertently malpositioned devices is a reportable sentinel event [5,6,7]. To mitigate this risk, chest radiography is commonly used to evaluate positioning after insertion, due to its wide availability and low cost. However, reports suggest that the accurate interpretation of device position on radiographs can be challenging [8,9,10], with reports from the UK’s National Health Service (NHS) indicating that more than half of serious incidents relating to malpositioned devices were related to the misinterpretation of post-insertion X-rays [4].

Deep learning, a subdomain of artificial intelligence (AI), enables effective outcome prediction and classification, and is influencing the optimisation and delivery of clinical medicine across specialties [11,12,13,14,15]. Developments in AI have the potential to improve clinician interpretation accuracy in radiology, as well as to automatically triage cases with suspected malpositioned catheters, shortening radiologist reporting turnaround times and improving the timeliness of patient care [11,16,17,18,19]. Historical solutions have relied on such rule-based approaches as edge detection, template matching, and morphological processing to detect the presence of ETTs and NGTs, or to classify their position [20,21]. More recently, deep convolutional neural network (DCNN) models have been used to predict ETT-carina distances, in order to recognise malpositioned ETT placement [22]. DCNNs that predict line position through a segmentation-based approach have also found use in the assessment of CVC tip positions and, by extension, the identification of malpositioned central lines [23,24]. Newer studies have examined DCNN algorithms capable of simultaneously assessing multiple types of lines and tubes [16,25,26,27,28]. Use of DCNN-based clinical decision support systems appears to improve chest X-ray (CXR) line detection accuracy and concordance amongst clinicians [29].

However, reported DCNN summary performance metrics may not translate to clinical practice. A recent systematic review identified recommended approaches for assessing DCNN performance, including determining the presence of a support device first, and subsequently the appropriateness of the device position [30]. As such, a more robust assessment of DCNN model performance involves providing an overall area under the receiver-operating characteristic curve (AUC) for the detection of the line/tube itself, and position classification AUC for only those cases where a line/tube is present. DCNN performance is usually summarised as a single metric (such as AUC) across an entire dataset, which may be misleading for clinically distinct and meaningful subgroups of patients in clinical practice, a phenomenon known as hidden stratification [31]. The classic example to illustrate hidden stratification is as follows. Algorithms designed to identify pneumothoraces with strong performance across an entire test dataset perform worse in subsets of patients without an accompanying intercostal drain, due to correlation between intercostal drains and pneumothoraces on CXR [32]. In the case of lines and tubes, performance discrepancies of the model across different subtypes of lines and tubes (such as jugular vs. subclavian central lines) may be hidden by the single summary metric.

Recently, a DCNN CXR tool capable of detecting 124 findings on frontal and lateral CXRs was developed by Seah and colleagues [16]. The model outperformed radiologists on 94% of findings and improved their diagnostic accuracy when assisting their interpretation for 80% of findings, including for lines and tubes. In the present study, which builds on the previous model performance evaluation study [16], we aimed to comprehensively assess the standalone performance of the DCNN algorithm for the identification of CVCs, ETTs, and NGTs, as well as their relevant device subtypes, on CXR. Our primary research question was: How does the algorithm perform when detecting clinically meaningful line and tube subcategories, and is it resilient to hidden stratification? Detection performance across the entire dedicated test set for CVCs, ETTs, and NGTs was calculated, as well as position classification performance with a full confusion matrix across cases known to contain the relevant device. Additionally, the performance of the DCNN across device subtypes was explored to assess the extent of hidden stratification. We hypothesised that model detection and positioning performance would not be degraded under this recharacterisation for CVCs, ETTs, and NGTs, and that the model would be resilient to hidden stratification.

## 2. Materials and Methods

### 2.1. Ethics Approval

This project was reviewed and approved by the Human Research Ethics Committee at the University of Notre Dame Australia (2020-127S, 21 September 2020). Data were deidentified prior to use in this study.

### 2.2. AI Model

The commercially available DCNN algorithm (Annalise CXR ver 2.0, Annalise-AI, Sydney, Australia) was evaluated. The deep-learning tool, described by Seah et al. (2021) [16], consisted of three DCNNs: an image projection classification model, a clinical finding classification model, and a clinical finding segmentation model. The image projection and classification models were based on the EfficientNet architecture [33]. The segmentation model was based on the U-Net [34] architecture with an EfficientNet backbone. For lines and tubes, the algorithm outputs consisted of both ‘satisfactory’ and ‘unsatisfactory’ findings, to evaluate positioning. A demonstration version of this algorithm is publicly accessible at https://cxrdemo.annalise.ai, accessed on 8 June 2023.

### 2.3. Study Data

The CXR test dataset was previously used to validate the DCNN algorithm and has been fully described elsewhere [16]. This test dataset was retrospectively assembled at the radiological study level from two sources: a large Australian private radiology company, as well as the publicly available MIMIC-CXR dataset [35]. Radiographic data was collected in a Digital Imaging and Communications in Medicine (DICOM) format with the original resolution and bit-depth preserved, and protected health information was removed through an automated deidentification process. Test dataset radiographs were selected only from patients on which the DCNN had not been trained. Inclusion criteria for this dataset were a patient age ≥ 16 years, and at least one frontal projection in the CXR study (PA or AP projections), corresponding to the intended use of the DCNN. The test dataset consisted of radiographs featuring the full spectrum of 124 findings that the DCNN algorithm was designed to detect, including pathology unrelated to lines and tubes (e.g., lung nodules and fractures). 

### 2.4. Ground Truth Labelling

Three Australian subspecialist thoracic radiologists (from a pool of seven) independently evaluated each case in the test dataset for the presence of any type of CVC, NGT, or ETT [16]. If at least one identified such a device, it was further ground-truth labelled for position and subtype, for the purposes of this study. Ground-truth labelling of line and tube position and subtype was performed by a thoracic subspecialist radiologist and an intensive care specialist. The ground truth was based on their consensus. The definitions for position and subtype were determined prior to commencement of ground truth labelling activities by a consensus discussion between the thoracic radiologist and intensivist, drawing from academic literature, guidelines, and clinical experience (Supplementary Table S1). Ground truth labelling activities were performed with access to the patient’s available past and future imaging, CXR reports with clinical information, as well as CT chest reports, if available. 

The ground truth labelling process of a line or tube position categorised its position as “satisfactory”, “suboptimal”, or “malpositioned”. Additionally, if an NGT was deemed to be incompletely/poorly imaged to the extent that position safety could not be reliably determined, this was classified as “incompletely imaged” by the ground truth labellers and was counted in the NGT detection metrics, but not counted in the position classification performance assessment. “Suboptimal” and “malpositioned” ground truth labels were combined into “Unsatisfactory” for this study, as the DCNN model was designed to group these categories together.

### 2.5. Analysis

#### 2.5.1. Primary Outcome

For each of the categories of CVC, ETT, and NGT, the AUC for detection of the presence of that device over the entire test dataset was calculated by bootstrap sampling the model performance over the test dataset 100,000 times, to derive both a mean and a 95% confidence interval (2.5th and 97.5th percentile). Then, for each of these three categories, separate “satisfactory position” and “unsatisfactory position” AUC performance scores were calculated over the subset of cases containing the relevant device as per the ground truth. The AUC has been reported as a mean and a 95% confidence interval by bootstrap sampling the test dataset 100,000 times over, before filtering that sample to retain only cases positive for that category, and then calculating the AUC on only those filtered cases for each iteration. Model outputs for position classifications were binarised using a previously derived “default” threshold, chosen based on the validation folds of the training data, then compared against the ground truth to derive the confusion matrix.

#### 2.5.2. Secondary Outcome

The position performance in subgroups of types of lines or tubes was analysed. Central lines were grouped into jugular, subclavian, dialysis, and peripherally inserted central catheters (PICCs). Enteric tubes were grouped into double lumen, NGTs with guidewires, and fine bore NGTs. Endotracheal tubes were grouped into true endotracheal tubes, and tracheostomies. To obtain position performance within a subgroup, bootstrap sampling the test dataset was again performed 100,000 times over, but each sample was filtered to retain only cases positive for that subgroup, before then calculating AUC performance. The obtained distribution of AUC values was analysed (as above) to obtain a mean and 95% confidence interval. This process was repeated for each position/subgroup combination. Again, model outputs were binarised using the predetermined threshold to derive a confusion matrix for the studies in that subgroup. Analyses were conducted using Python, using the SciPy [36], Scikit-learn [37], NumPy [38], and Tensorflow [39] packages. Results were independently calculated and agreed upon by two investigators.

## 3. Results

The analysed dataset contained 2568 studies with 4568 images, representing 2286 patients. Forty-three percent of cases from the test dataset originated from the MIMIC-CXR dataset and 57 percent originated from the private Australian radiology practice dataset. Table 1 presents the demographic and imaging characteristics of the test dataset. 

Eleven of the studies were deemed unsuitable for DCNN processing by the system for technical reasons (e.g., where no frontal image was recognised using the model, no CXR image was found using the model, where a processing error occurred, or case data were missing) and were excluded. Four excluded studies contained a line or tube. One contained a satisfactory PICC, two contained a satisfactory subclavian line, and one contained both a satisfactory jugular line and an unsatisfactory fine bore NGT. 

In the remaining 2557 studies, there were 751 cases containing a line or tube of interest: 477 cases contained a CVC, 262 cases contained an ETT, and 206 cases contained an NGT that were not incompletely imaged. Thirty-one NGTs were incompletely imaged, and a position determination could not be confidently assigned by the ground truth labellers, and were thus excluded from the position classification analysis. NGTs with wires in situ were not analysed as a subgroup for position classification as there were too few cases. Tracheostomies as a subgroup were not analysed for AUC as only one result (“satisfactory”) was seen in that subgroup.

### 3.1. Primary Outcome

**Figure 1:**
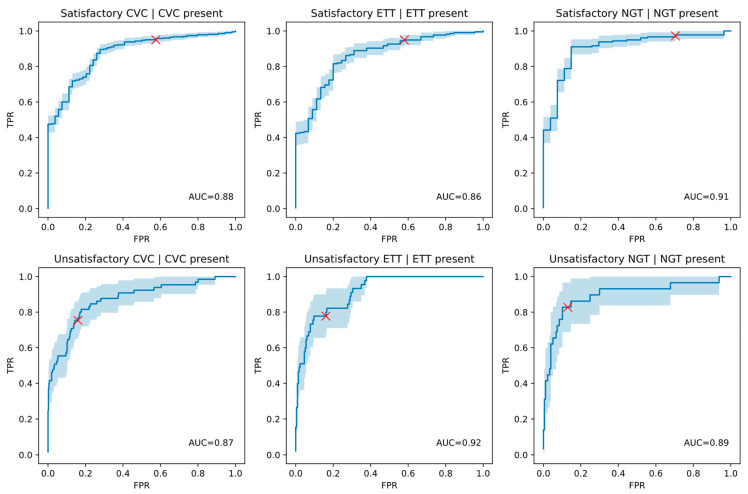
AUC curves for the position classification performance over ground truth positive cases only, with the operating point at the default-threshold indicated with a red cross for each finding. A 95% confidence interval for the AUC curve is shaded in light blue.

The DCNN model identified the presence of ETTs, NGTs, and CVC with AUCs greater than 0.99 (Table 2). The mean satisfactory and unsatisfactory position determination AUC performance of the DCNN over the relevant category of line or tube ranged from 0.86 to 0.91 for the six findings (Table 3), with the relatively wide 95% confidence intervals. The size of the category over which performance was calculated ranged from 206 to 477 cases. The confusion matrix for each of the findings has been shown using the default threshold supplied with the model. AUC curves for position classification performance over ground truth positive cases have been shown in Figure 1, with the operating point at the preselected threshold indicated.

### 3.2. Secondary Outcome

Eighteen subgroup analyses over nine line/tube subtypes were performed for the secondary outcome to define model position classification performance, with results shown in Table 4. The size of the subpopulation over which performance was calculated ranged from 37 to 243 (Table 4). A confusion matrix using the default threshold supplied with the model is displayed in Table 4. The position classification performance across subgroups ranged from 0.79 to 1.00, with notable variability between different CVC subtypes with the lowest AUC performance in the PICC subgroup. Tracheostomy position performance could not be calculated as the test dataset contained only satisfactory tracheostomies.

## 4. Discussion

This study involved a detailed performance analysis and substratification in the context of an established CXR deep learning model reviewing a post-insertion CXR for the satisfactory/unsatisfactory positioning of a line or tube. Existing research has tended to describe model performance over entire mixed datasets (including cases with and without devices). On such datasets, one DCNN algorithm was reported to achieve AUCs for NGT position classification of 0.82 to 0.87, and another achieved AUCs for low vs. normal ETT position of 0.74 to 0.81 [40,41]. A newer multifinding algorithm demonstrated an AUC for detection of unsatisfactory ETT, CVC, and NGT of 0.919, 0.769, and 0.931, respectively, across a mixed test dataset of 70,209 images [26]. In this study, model performance for detection of lines and tubes over the entire test dataset was high. AUCs exceeded 0.99 for the three device categories, and position classification performance demonstrated that the model still performed favourably with AUCs from 0.86 to 0.91 across the cases containing that category of device. Position classification performance of the DCNN only over the subset of cases that were ground-truth labelled as containing that device was expected to be lower than position performance over the entire dataset (previously presented in Seah et al., 2021 [16]), as whole dataset performance is inflated by the fact that it correctly identifies the absence of the line/tube in the large number of the negative cases. Overall, the DCNN algorithm analysed herein displays position classification performance over ground truth positive cases comparable to published mixed dataset performance of other algorithms.

**Figure 2:**
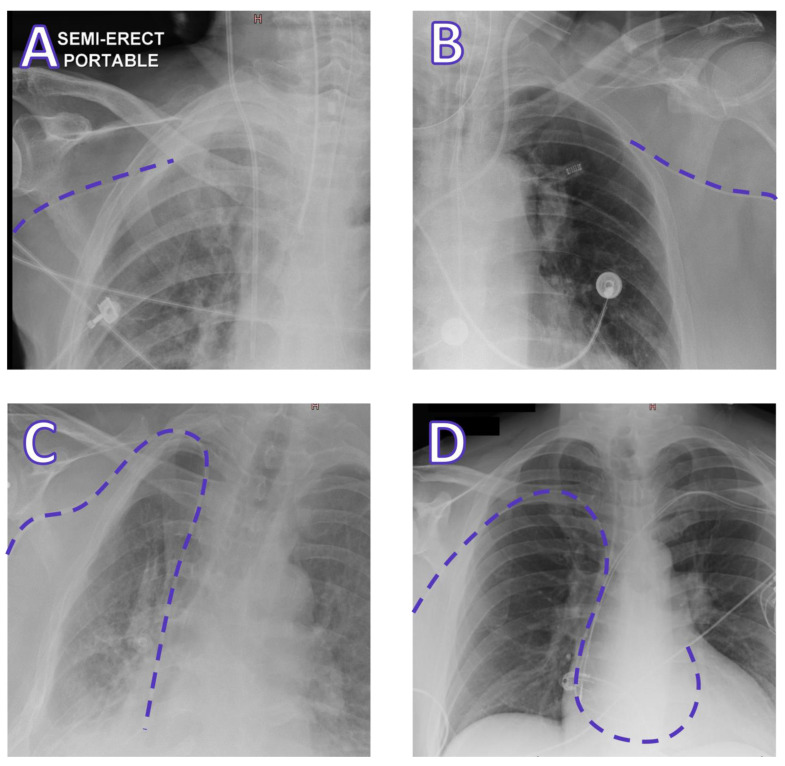
Representative examples of false negative malpositioned PICCs, with paths highlighted by dashed lines: (**A**) A right-sided PICC terminating in the subclavian vein; (**B**) A left-sided PICC terminating in the subclavian vein; (**C**) A right-sided PICC with the tip in the proximal right atrium; and (**D**) A brachial Swan-Ganz catheter with tip in the right-ventricular outflow tract. These are some examples of malpositioned lines that were not classified by the model as being malpositioned. Cases are from a test dataset containing radiographs from a private Australian radiology practice and the MIMIC-CXR dataset [35].

Model performance was mostly resilient to hidden stratification across subtypes of lines and tubes. However, PICC position classification performance appeared to be degraded compared to other CVC subtypes, representing the majority of false negative (FN) unsatisfactory CVCs, suggesting a potential subgroup subject to hidden stratification. We hypothesise that this is because PICCs are thinner and more difficult to visualise compared to the thicker jugular, subclavian, and dialysis lines, and that the model has likely become attuned to tip positions centrally (both satisfactory and unsatisfactory) due to the abundance of examples, resulting in a degraded performance for peripherally located tips that are rarer and more variable in location. PICCs terminating near the axilla represented the majority of FN unsatisfactory PICCs. Figure 2 provides a collection of examples of FN malpositioned PICCs. Investigations using datasets enriched with these examples are required to further characterise this behaviour. 

The finding of an unsatisfactory device being predicted as satisfactory (FN) by the model is clinically more consequential than a satisfactory device predicted as unsatisfactory (FP). As such, the occurrence of these was further defined. There were sixteen cases of FN unsatisfactory CVCs: three malpositioned CVCs, and thirteen suboptimal CVCs. Of the three FN malpositioned CVCs, one was misclassified by the model as “satisfactory”, namely, a left-sided PICC terminating in the right subclavian. The two other FN malpositioned CVCs were not misclassified as “satisfactory” but were simply missed by the model; these were a left-sided jugular line looping back up the ipsilateral internal jugular vein, and a left-sided subclavian line deviating into the ipsilateral internal jugular vein. Such malpositioned lines represent rare cases that are challenging for AI models to interpret due to their low prevalence in training datasets. Rarer devices were also often misinterpreted, such as a right-sided brachial Swan-Ganz catheter with the tip in the right ventricular outflow tract. More common cases, such as suboptimal lines with tips in the right atrium, were better identified, but still represented five of the FN unsatisfactory CVC cases. 

There were ten cases of FN unsatisfactory ETTs, all meeting the ‘suboptimal’ definition, and no cases of missed ETT malposition within a main bronchus. Of these ten cases, four demonstrated tube tips between 20 and 30 mm from the carina, five were between 70 and 85 mm from the carina, and the remaining case contained a tube 100 mm from the carina but also contained an endoscope located in the oesophagus. All ten cases had an enteral device (nine NGTs and one endoscope), suggesting that the model may suffer from hidden stratification of ETT position classification in cases containing such an accompanying device, compared to cases without one. There was insufficient prevalence in the testing dataset of rarer oesophageal devices, such as endoscopes or transoesophageal echocardiogram probes, to investigate whether these negatively affected ETT position assessment performance. 

There were five cases of FN unsatisfactory NGTs. None demonstrated the model missing a malpositioned enteric tube in the airways. One study contained two images, the first with an oesophageal malpositioned NGT, and the second with the tip adequately in the stomach, presumably after advancement, which is what likely led to the misinterpretation by the model, which produces predictions on a per-study basis. Another study demonstrated an ETT overlying a malpositioned oesophageal NGT on the radiograph, resulting in the NGT being undetected by the model; this represents a clinically important subpopulation of malpositioned NGTs with poor tip visibility that appears to be challenging for AI models. The remaining three cases demonstrated incompletely imaged NGTs either due to acquisition parameters, or obscuration from additional devices. Two malpositioned cases with subdiaphragmatic NGTs with the proximal hole residing above the gastro-oesophageal junction were misclassified as satisfactory. AI models appear to struggle in these subpopulations, especially if they have not been explicitly trained to interpret incompletely imaged studies, as position assessment is often difficult or inconclusive in these cases.

This study had several limitations. Firstly, datasets enriched with further examples of rarer cases (such as malpositioned fine bore NGTs, malpositioned tracheostomies, and malpositioned dialysis catheters, as well as identified subgroups of interest such as malpositioned NGTs with ETTs, oesophageal devices, and PICCs with axillary tips) are needed to elucidate model performance in these scenarios. Secondly, although this analysis was carried out on a held-out test dataset, this did not represent an external dataset, as the test dataset (while exclusive at the patient level) was drawn from some of the same sources as the training data. It has been widely reported that diagnostic accuracy of models may decrease when applied to external datasets originating from sites that did not contribute to the training dataset [42,43]. Finally, this study represents a retrospective in vitro analysis, and studies (retrospective or prospective) to determine the clinical effects of critical care clinicians using the AI device to assess line and tube positions are needed, to evaluate their real-world performance [44].

## 5. Conclusions

There is a general need in the literature for a more comprehensive and detailed approach to describing the performance of line/tube position classification algorithms. Here, we presented the results of an indepth analysis investigating the performance of the DCNN algorithm developed by Seah et al. [16]. This DCNN displayed high detection performance and good position classification performance for CVCs, ETTs, and NGTs, supporting its use as an AI-assistive device. There was a small number of misinterpreted cases. Subgroup analysis identified potential hidden stratification for PICC lines, as well as in cases of ETT with an accompanying enteral device. This illustrates that, despite strong DCNN performance overall, more detailed analysis of device performance is necessary to evaluate for hidden stratification. Further work is recommended to investigate model performance in clinically relevant line and tube subtypes, especially central lines, as well as specific edge-case scenarios that are encountered in radiological practice. This study highlights the continuing need for radiological studies to be reviewed by clinicians in conjunction with DCNN models to achieve optimal interpretation outcomes.

## Figures and Tables

**Table 1:** Demographics of the overall test dataset. * MIMIC-CXR does not provide sex or age information.

Dataset Characteristic	Statistics
Patients	2286
Studies	2568
Images	4568
Sex	29% male 28% female 43% unknown *
Age	74 years (SD 15 years) *
View Position	28% PA 33% AP 31% LAT 8% other

**Table 2:** Detection performance of the model over the entire testing dataset of 2557 processed studies. The mean AUC of the model and its nonparametric 95% confidence interval from 100,000 bootstrap iterations are presented. Positives and negatives represent the number of cases with and without the finding, respectively, in the dataset.

Finding	Positives	Negatives	Model AUC Mean over Entire Dataset	AUC Mean 95% CI
ETT	262	2295	0.9999	0.9997–1.0000
CVC	477	2080	0.9983	0.9970–0.9993
NGT	206	2351	0.9994	0.9984–1.0000

**Table 3:** Position classification performance of the model over the ground-truth positive cases of the relevant line/tube (‘Subpopulation’). The mean AUC of the model and its nonparametric 95% confidence interval from 100,000 bootstrap iterations are presented, along with a confusion matrix for the classification of the cases based on the default threshold selected for the model. The subgroup size =/= positive case in subgroup for CVC and NGT because they can have multiple lines/tubes per patient. TN = True negative. FP = False positive. FN = False negative. TP = True positive.

Finding	Category	Size of Category	Positive Cases in Category	Model AUC Mean (95% CI)	TN	FP	FN	TP
Satisfactory ETT	ETT	262	209	0.8608 (0.8020–0.9128)	19	34	11	198
Unsatisfactory ETT	ETT	262	53	0.9153 (0.8724–0.9519)	174	35	18	35
Satisfactory CVC	CVC	477	423	0.8778 (0.8323–0.9186)	23	31	21	402
Unsatisfactory CVC	CVC	477	65	0.8715 (0.8158–0.9200)	346	66	16	49
Satisfactory NGT	NGT	206	179	0.9051 (0.8409–0.9574)	8	19	5	174
Unsatisfactory NGT	NGT	206	29	0.8943 (0.8062–0.9620)	154	23	5	24

**Table 4:** Position classification performance of the model over ground truth cases containing a certain subtype of line/tube (‘Subgroup). The number of cases in that subgroup and the number of positives for the finding are shown. The mean AUC of the model and its nonparametric 95% confidence interval from 100,000 bootstrap iterations are presented, along with a confusion matrix for the classification of the cases based on the default threshold selected for the model. The subgroup size may not equal positive cases in subgroup for CVC and NGT because they can have multiple lines/tubes per patient. TN = True negative. FP = False positive. FN = False negative. TP = True positive. * No AUC could be calculated as there were no unsatisfactory tracheostomies in the test dataset. ^†^ Too few cases were present in the subgroup for meaningful AUC calculation.

Finding	Subgroup	Size of Subgroup	Positive Cases in Subgroup	Model AUC Mean (95% CI)	TN	FP	FN	TP
Satisfactory CVC	Dialysis Catheters	40	36	0.9304 (0.8222–1.0000)	1	3	2	34
Unsatisfactory CVC	Dialysis Catheters	40	6	0.9019 (0.6989–1.0000)	24	10	1	5
Satisfactory CVC	Jugular Lines	243	221	0.9139 (0.8509–0.9639)	10	12	6	215
Unsatisfactory CVC	Jugular Lines	243	32	0.8700 (0.7890–0.9379)	176	35	7	25
Satisfactory CVC	PICCs	140	123	0.8227 (0.7220–0.9083)	7	10	13	110
Unsatisfactory CVC	PICCs	140	23	0.7880 (0.6624–0.8947)	98	19	9	14
Satisfactory CVC	Subclavian Lines	121	107	0.8879 (0.7943–0.9639)	5	9	2	105
Unsatisfactory CVC	Subclavian Lines	121	16	0.8892 (0.7840–0.9675)	87	18	4	12
Satisfactory ETT	Endotracheal Tubes	211	166	0.8709 (0.8130–0.9220)	19	26	8	158
Unsatisfactory ETT	Endotracheal Tubes	211	45	0.8923 (0.8387–0.9384)	131	35	10	35
Satisfactory ETT	Tracheostomies	51	51	N/A *	0	0	3	48
Unsatisfactory ETT	Tracheostomies	51	0	N/A *	51	0	0	0
Satisfactory NGT	Double Lumen NGTs	170	147	0.9091 (0.8388–0.9655)	7	16	3	144
Unsatisfactory NGT	Double Lumen NGTs	170	25	0.8752 (0.7741–0.9538)	125	20	5	20
Satisfactory NGT	NGTs with Guide Wire	2	1	N/A ^†^	1	0	0	1
Unsatisfactory NGT	NGTs with Guide Wire	2	1	N/A ^†^	1	0	0	1
Satisfactory NGT	Fine Bore NGTs	37	33	0.9091 (0.8000–1.0000)	1	3	2	31
Unsatisfactory NGT	Fine Bore NGTs	37	4	1.0000 (1.0000–1.0000)	30	3	0	4

## Data Availability

Data are available on reasonable request. The research team may make the model and radiologist performance datasets and the test dataset available to interested research partners with the goals of supporting the research community and making further collaborative contributions to the literature. Requests for access can be made through the Annalise.ai website (https://annalise.ai/contact/, accessed on 8 June 2023). The model is publicly available as a commercial software product (https://annalise.ai/products/annalise-cxr/, accessed on 8 June 2023). The free web-based demonstration can be accessed online (https://cxrdemo.annalise.ai/, accessed on 8 June 2023).

## Supplementary Materials

The following supporting information can be downloaded at: https://www.mdpi.com/article/10.3390/diagnostics13142317/s1, Supplementary Table S1: Position definitions.
