# Supplementary material for: Analysis of Line and Tube Detection Performance of a Chest X-ray Deep Learning Model to Evaluate Hidden Stratification
Source: Diagnostics (Basel). 2023 Jul 9;13(14):2317. doi: 10.3390/diagnostics13142317 (PMC10378683; doi:10.3390/diagnostics13142317)
Supplement: Supplementary file 1 [file diagnostics-13-02317-s001.zip › diagnostics-2469572-supplementary.pdf]

Supplementary Table S1: Position definitions for the lines and tubes subtypes included in this study. Findings considered to be "Suboptimal" and "malpositioned were combined into a single "Unsatisfactory" finding for this study.

**Line/Tube Subtype**	**Position Assessment**		
	**Satisfactory**	**Suboptimal**	**Malpositioned**
**Endotracheal Tubes**	All the following must be present:<br>• The tube tip lies within the trachea.<br>• The tip lies between 3cm and 7cm from the carina.	All the following must be present:<br>• The tube tip lies within the trachea.<br>• The tip lies either <3cm from the carina OR >7cm from the carina.	The tube tip lies outside the trachea. Either/or:<br>• Non-bronchial<br>• Bronchus position<br>o Right<br>o Left
**Tracheostomies**	The tube tip lies within the trachea more than 1cm above the carina.	The tracheostomy tube tip is less than 1cm from the carina.	The tube tip lies outside the trachea.
**Nasogastric Tubes (all subtypes)**	All the following must be present:<br>• Tip of tube is in the stomach, below the diaphragm, and if a side hole is present, it is also clearly within the stomach.<br>• At least 8 cm of the tube is visible beyond the gastro-esophageal junction.<br>• No looping of the tube above the gastro-esophageal junction.	All the following must be present:<br>• Tip of tube is below the diaphragm, however there is less than 8cm beyond the gastro-esophageal junction OR the tip is projected over the proximal small bowel.<br>• Any visible side hole must be below the diaphragm.<br>• No looping of the tube above the gastro-esophageal junction.	One or more of the following must be present:<br>• The tube runs outside of the gastrointestinal tract (e.g., down the trachea).<br>• The tip of the tube is within the oropharynx, hyopharynx, larynx, esophagus, or the side hole is above the gastro-esophageal junction.<br>• Looping of the line in the esophagus or upper airways.
**Non-Dialysis Jugular and Subclavian Central Lines**	All the following must be present:<br>• Line has no loops.<br>• Tip terminates within the desired pathway from insertion to the cavoatrial junction.<br>• Visible side holes are satisfactory in position.	All the following must be present:<br>• Line has no loops.<br>• Tip traverses the desired pathway from insertion to the cavoatrial junction and terminates in the right atrium.<br>• Visible side holes are satisfactory in position.	One or more of the following must be present:<br>• Catheter looped.<br>• Catheter terminates outside the desired pathway, including but not limited to malposition into the azygos vein, contralateral jugular or subclavian vein, left or right axillary vein, right ventricle, pulmonary artery, inferior vena cava.<br>• The catheter is discontinuous indicating fracture and/or migration.<br>• Visible side holes are not satisfactory in position.
**Dialysis Catheters**	All the following must be present:<br>• Line has no loops.<br>• Tip terminates within the desired pathway from insertion to the cavoatrial junction.<br>• Visible side holes are satisfactory in position.	All of the following must be present:<br>• Line has no loops.<br>• Tip terminates in the right atrium OR abuts the lateral wall of the SVC at a perpendicular angle.<br>• Visible side holes are satisfactory in position.	One or more of the following must be present:<br>• Catheter looped.<br>• Catheter terminates outside the desired pathway, including but not limited to malposition into the azygos vein, left or right jugular veins, right subclavian vein, left or right axillary vein, right ventricle, pulmonary artery, inferior vena cava.<br>• The catheter is discontinuous indicating fracture and/or migration.

			• Visible side holes are not satisfactory in position.
**PICCs**	All the following must be present:<br>• Line has no loops.<br>• Tip terminates within the desired pathway from insertion to the cavoatrial junction and has crossed the anterior first ipsilateral rib.	All the following must be present:<br>• Line has no loops.<br>• Tip terminates within the desired pathway from insertion to the cavoatrial junction and has not crossed the ipsilateral anterior first rib OR is in the right atrium.	One of the following must be present:<br>• Catheter looped.<br>• Catheter terminates outside the desired pathway, including but not limited to malposition into the azygos vein, jugular vein, contralateral innominate vein, right ventricle, pulmonary artery, inferior vena cava.<br>• The catheter is discontinuous indicating fracture and/or migration.
